# Quality of life impact and recovery after ureteroscopy and stent insertion: insights from daily surveys in STENTS

**DOI:** 10.1186/s12894-022-01004-9

**Published:** 2022-04-06

**Authors:** Jonathan D. Harper, Alana C. Desai, Jodi A. Antonelli, Gregory E. Tasian, Justin B. Ziemba, Hussein R. Al-Khalidi, H. Henry Lai, Naim M. Maalouf, Peter P. Reese, Hunter B. Wessells, Ziya Kirkali, Charles D. Scales, NIDDK Urinary Stone Disease Research Network (USDRN)

**Affiliations:** 1 Department of Urology, University of Washington School of Medicine, Seattle, WA 98195 USA; 2 Division of Urologic Surgery, Department of Surgery, Washington University School of Medicine, St Louis, MO USA; 3 Department of Urology, University of Texas Southwestern Medical Center, Dallas, TX USA; 4 Division of Pediatric Urology, Department of Surgery, The Children’s Hospital of Philadelphia, Philadelphia, PA USA; 5 Division of Urology, Department of Surgery, Hospital of the University of Pennsylvania, Philadelphia, PA USA; 6 Department of Biostatistics and Bioinformatics, Duke Clinical Research Institute, Duke University School of Medicine, Durham, NC USA; 7 Department of Anesthesiology, Washington University School of Medicine, St Louis, MO USA; 8 Department of Internal Medicine, Charles and Jane Pak Center for Mineral Metabolism and Clinical Research, University of Texas Southwestern Medical Center, Dallas, TX USA; 9 Renal-Electrolyte and Hypertension Division, Department of Biostatistics, Epidemiology, and Informatics, Perelman School of Medicine, University of Pennsylvania, Philadelphia, PA USA; 10 Department of Biostatistics, Epidemiology and Informatics, Perelman School of Medicine, University of Pennsylvania, Philadelphia, PA USA; 11 National Institute of Diabetes and Digestive and Kidney Diseases, Bethesda, MD USA; 12 Departments of Surgery and Population Health Science, Duke Surgical Center for Outcomes Research, Duke Clinical Research Institute, Duke University School of Medicine, Durham, NC USA

**Keywords:** Urinary stone disease, Ureteroscopy, Ureteral stent, Stent-associated symptoms

## Abstract

**Background:**

Our objective was to describe day-to-day evolution and variations in patient-reported stent-associated symptoms (SAS) in the STudy to Enhance uNderstanding of sTent-associated Symptoms (STENTS), a prospective multicenter observational cohort study, using multiple instruments with conceptual overlap in various domains.

**Methods:**

In a nested cohort of the STENTS study, the initial 40 participants having unilateral ureteroscopy (URS) and stent placement underwent daily assessment of self-reported measures using the Brief Pain Inventory short form, Patient-Reported Outcome Measurement Information System measures for pain severity and pain interference, the Urinary Score of the Ureteral Stent Symptom Questionnaire, and Symptoms of Lower Urinary Tract Dysfunction Research Network Symptom Index. Pain intensity, pain interference, urinary symptoms, and bother were obtained preoperatively, daily until stent removal, and at postoperative day (POD) 30.

**Results:**

The median age was 44 years (IQR 29,58), and 53% were female. The size of the dominant stone was 7.5 mm (IQR 5,11), and 50% were located in the kidney. There was consistency among instruments assessing similar concepts. Pain intensity and urinary symptoms increased from baseline to POD 1 with apparent peaks in the first 2 days, remained elevated with stent in situ, and varied widely among individuals. Interference due to pain, and bother due to urinary symptoms, likewise demonstrated high individual variability.

**Conclusions:**

This first study investigating daily SAS allows for a more in-depth look at the lived experience after URS and the impact on quality of life. Different instruments measuring pain intensity, pain interference, and urinary symptoms produced consistent assessments of patients’ experiences. The overall daily stability of pain and urinary symptoms after URS was also marked by high patient-level variation, suggesting an opportunity to identify characteristics associated with severe SAS after URS.

**Supplementary Information:**

The online version contains supplementary material available at 10.1186/s12894-022-01004-9.

## Introduction

Patients who receive a ureteral stent often experience debilitating symptoms, including pain, urinary urgency and frequency, hematuria, and incontinence. Researchers have struggled to understand and predict the severity and range of stent-associated symptoms (SAS) following ureteroscopy (URS) for stone disease. While most patients will have some degree of SAS, it is unknown who will suffer from severe symptoms that significantly affect quality of life. Furthermore, a clear understanding of the daily variation of SAS after URS remains elusive; having this information is critical to patient counseling, serving as a building block for stent-related research, and employing strategies to mitigate these symptoms.

Investigation of SAS is a research priority as URS has become the most common procedure performed for renal and ureteral stones, and stents are used in most cases [1–3]. Contrary to popular belief, some studies have shown that stents are not associated with increased hospital returns [4] and may actually reduce unplanned visits [3, 5, 6]. Therefore, despite SAS, ureteral stents continue to play an important role in reducing complications such as urinary obstruction, and may slightly reduce ureteral stricture formation [5]. Given the limited insight into drivers of SAS and lack of characterization of the patient’s daily experience after URS, the Urinary Stone Disease Research Network is conducting the STudy to Enhance uNderstanding of sTent-associated Symptoms (STENTS), a prospective observational cohort study.

A key consideration for improving understanding of SAS is assessment of patient-reported symptoms. Patient-reported measures provide important information about the impact of a condition and/or treatment from the patient’s perspective. Within the overall STENTS study, an initial nested cohort study was designed as a unique opportunity to gain a better understanding of patients’ daily experiences after URS for stone treatment. Our goal was to determine the trajectory of day-to-day variations in pain intensity, pain interference, urinary symptoms, and bother, identify a peak day of symptoms, and provide guidance for future studies in choosing outcome measures.

## Materials and methods

### Study design and nested cohort

STENTS is a multi-institutional prospective observational cohort study of individuals undergoing URS and ureteral stent placement for treatment of a ureteral or renal stone. A complete description of the STENTS protocol has been published [7]. The initial 40 participants made up the nested cohort. A minimum number of participants based on age and gender were prespecified to ensure inclusion of specific age and gender groups in this nested cohort: the minima required were 8 individuals aged 12–25, 12 males aged > 25 years, and 12 females aged > 25 years.

In brief, the nested cohort study was designed such that participants would complete all study procedures performed in the subsequent larger STENTS observational cohort. Unique to the nested cohort, these 40 participants completed daily questionnaires that assessed pain intensity, pain interference, urinary symptoms, and bother, and also participated in a semi-structured interview to further characterize the patient experience. The prespecified objectives of the nested cohort were to generate knowledge about the daily variation of pain intensity, pain interference, urinary symptoms, and bother; identify a peak day of symptoms; and evaluate overlap in experience assessment among the instruments. These analyses informed the selection of instruments and the timing of their administration for the main STENTS cohort.

### Study population

Individuals aged 12 and older with a planned unilateral URS for stone treatment were recruited from four clinical centers. All participants were prospectively enrolled after institutional review board approval. Participants aged 17 and under provided their informed assent, and their parents provided parental permission. Exclusion criteria were an indwelling ureteral stent, receipt of a stent in the preceding 60 days, concomitant shockwave lithotripsy or percutaneous nephrolithotomy, conditions resulting in neurogenic bladder dysfunction, anatomic urological abnormality resulting in abnormal bladder sensation, or renal transplantation; bedridden and vulnerable populations were also excluded.

### Study procedures

Participants completed baseline questionnaires that recorded individual characteristics, medical and stone history, and medication use. Participants with a history of a ureteral stent reported whether they had severe pain or urinary symptoms with their prior stent. Intraoperative data, including stone features, details of ureteral instrumentation, irrigation type, and stent characteristics, were prospectively collected at the time of URS.

### Patient experiences

All participants in the nested STENTS cohort were administered the following questionnaires that assessed pain, urinary symptoms, and the manner and degree to which these symptoms impact the patient’s life (interference and bother): (1) the Brief Pain Inventory (BPI) short form [8], which has been used in pain studies widely, assessed pain intensity and pain interference; (2) Patient-Reported Outcome Measurement Information System (PROMIS) [9] measures of pain intensity and pain interference allowed for comparing scores to population norms; (3) the Urinary Score of the Ureteral Stent Symptom Questionnaire (USSQ) [10], an instrument developed for SAS over a 1-month recall period, assessed urinary symptoms and bother; and (4) Symptoms of Lower Urinary Tract Dysfunction Research Network Symptom Index (LURN SI-10) assessed urinary symptoms and bother [11]. We intentionally administered questionnaires that assessed the same construct in order to determine potential differences in the measured experience. We acknowledge that the USSQ covers other domains besides urinary symptoms, but given the importance of characterizing pain intensity and pain interference as accurately as possible, being able to compare to population norms, and our desire for a comprehensive body map that incudes genitalia, it was decided to incorporate specific pain instruments that are used in the broader community of medicine. This was after several discussions with a multidisciplinary field including experts within pain medicine, pain psychology, and psychometricians well versed in development of patient reported outcome measures. Table 1 shows the instruments listed by SAS domain.

**Table 1:** Self-reported measures used in the study by stent-associated symptoms domain

Instrument	Domain			
	Pain Intensity	Pain Interference	Urinary Symptoms	Urinary Bother
BPI	•	•		
LURN SI-10			•	•
PROMIS	•	•		
USSQ-U			•	•

BPI = Brief Pain Inventory; LURN SI-10 = Symptoms of Lower Urinary Tract Dysfunction Research Network Symptom Index; PROMIS = Patient-Reported Outcome Measurement Information System; USSQ-U = urinary score of the Ureteral Stent Symptom Questionnaire

### Data collection

Participants completed the above questionnaires preoperatively (baseline), on postoperative day (POD) 1, daily until stent removal, including day of stent removal, and 30 days after stent removal (Table 1). Questionnaires were self-administered and completed each day via electronic format, or paper copies if preferred. Participants received an electronic link each day reminding them to complete the forms.

Participants were contacted by study staff to record any adverse events. Prescribed medications following surgery were assessed using a medication diary. Finally, participants were asked about any health care utilization during the 30 days following stent removal.

### Statistical analysis

Data are summarized as medians (25th, 75th percentiles) and means (SDs) for continuous variables and as counts (percentages) for categorical variables. Due to multiple outcomes assessed at many time points and the small sample size, we did not test for differences in symptom severity across instruments or days. Summary statistics of daily patient-reported symptoms were calculated, and data are graphically depicted in box-and-whisker plots. All statistical summary data were generated using SAS statistical software version 9.4 (SAS Institute, Inc., Cary, NC).

## Results

Forty participants were included, comprising 9 individuals (3 male and 6 female) aged 12–25 years, 16 males aged > 25 years, and 15 females aged > 25 years. The median age was 44 years (IQR 29, 58), and 53% were female. Over half (58%) of the participants had a prior history of kidney stones. Of the 13 (33%) who had a ureteral stent in the past, 8 reported having severe pain and 6 reported having severe urinary symptoms with their previous stent. Participant characteristics are listed in Table 2, and intraoperative data are shown in Table 3. Most participants had more than one stone treated, with the dominant stone location evenly split between renal and ureteral. Stone sizes and location represent stones treated during surgery.

**Table 2:** Participant characteristics at baseline

Characteristic	Participants (n = 40)
Sex: female	21 (53%)
Race	
White	38 (95%)
Black	2 (5%)
Ethnicity: Hispanic/Latinx	3 (8%)
Age (years)	
Median (IQR)	43.5 (28.5, 57.5)
Mean (SD)	43.1 (17.6)
Medical history	
Depression	9 (23%)
Anxiety	8 (20%)
Mood disorder (other)	1 (3%)
Chronic pain condition	7 (18%)
Previous stone history	23 (58%)
Prior ureteroscopy	12 (30%)
Prior ureteral stent placement	13 (33%)
Severe pain with prior ureteral stent	8 (20%)
Severe urinary symptoms with prior ureteral stent	6 (15%)
Medication use in past 30 days	
Opioids	11 (28%)
NSAIDs	20 (50%)
Tamsulosin (or alpha blocker)	16 (40%)
Oxybutynin (or anticholinergic)	3 (8%)

Data shown are n (%) except where indicated. IQR = 25th, 75th percentiles; SD = standard deviation; NSAIDs = nonsteroidal anti-inflammatory drugs

**Table 3:** Intraoperative data

Variable	Participants (n = 40)
Side of treatment	
Right	19 (48%)
Left	21 (52%)
Dominant stone size (renal, mm)	
Median (IQR)	7.5 (5.0, 10.5)
Mean (SD)	7.5 (3.9)
Dominant stone size (ureteral, mm)	
Median (IQR)	6.0 (5.0, 7.0)
Mean (SD)	6.2 (2.2)
Dominant stone location*	
Renal	20 (50%)
Ureter (proximal)	7 (18%)
Ureter (distal)	13 (33%)
Number of stones treated (renal)	
Median (IQR)	2 (1, 4)
Mean (SD)	5.6 (10.6)
Number of stones treated (ureteral)	
Median (IQR)	1 (1, 2)
Mean (SD)	1.3 (0.5)
Operative time (min)	
Median (IQR)	51 (36, 80)
Mean (SD)	59.1 (28.6)
Ureteroscopy time (min)	
Median (IQR)	32.5 (20, 58.5)
Mean (SD)	40.1 (26.3)
Ureteroscope type*	
Flexible	24 (60%)
Semirigid	7 (18%)
Both	9 (23%)
Ureteral access sheath use	19 (48%)
Basket extraction	33 (82%)
Irrigation	
Manual	20 (50%)
Constant pressure	20 (50%)
Ureteral stent diameter	
4.7 French	6 (15%)
6 French	34 (85%)
Ureteral stent length* (cm)	
22	1 (3%)
24	18 (45%)
26	14 (35%)
28	5 (13%)
30	2 (5%)
Stent dwell time (days)	
Median (IQR)	8 (6.5, 11)
Mean (SD)	10.1 (6.8)

*Note: percentages may not sum to 100 due to rounding

Data shown are n (%) except where indicated

IQR = 25th, 75th percentiles; SD = standard deviation

**Fig. 1:**
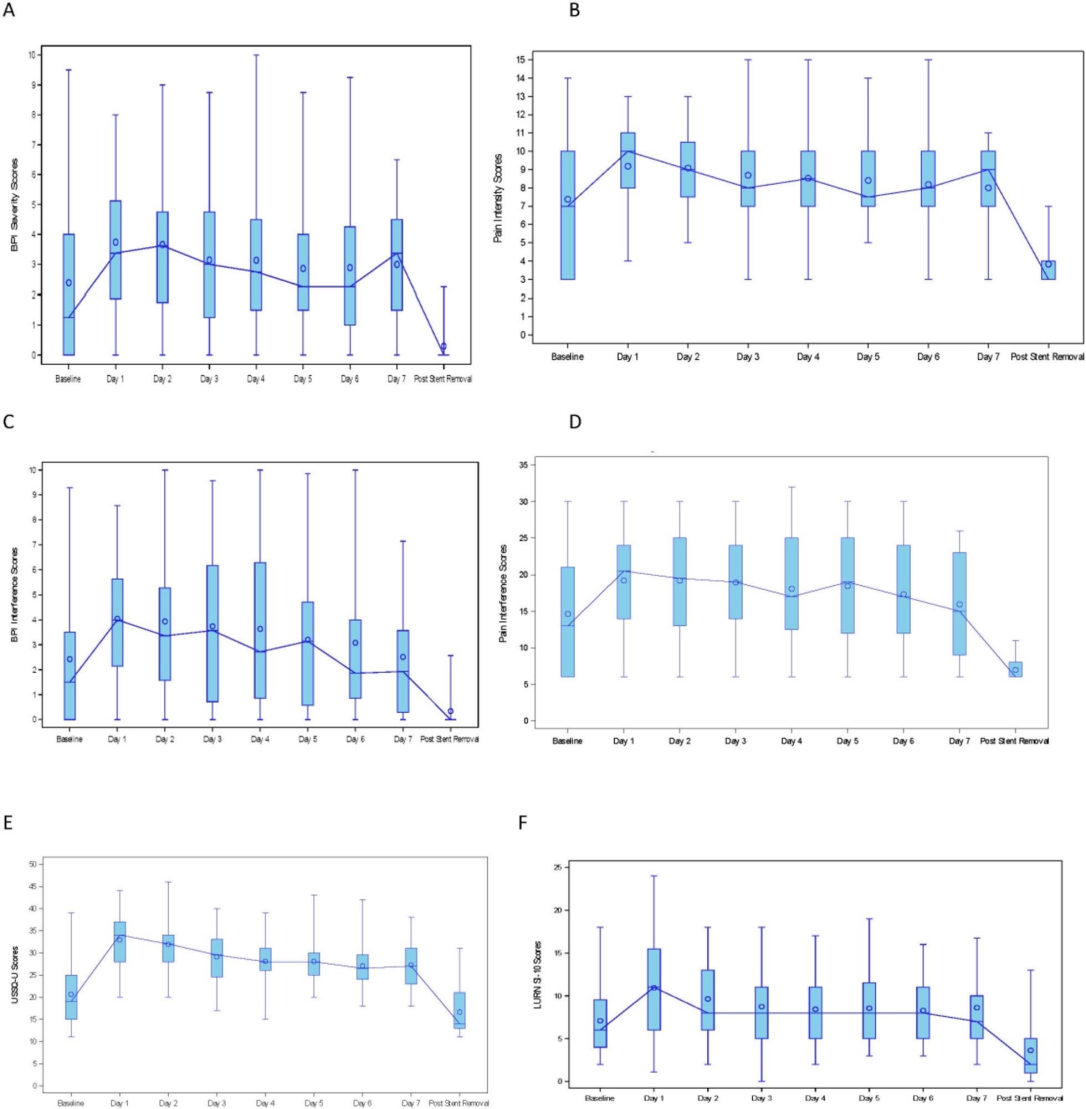
Daily stent-associated symptoms in each domain measured with various instruments displayed in box and whisker plots. The range (whiskers) is shown for each timepoint while the box represents the interquartile range. Circles depict mean values and the median values are connected by a line: **A** BPI pain severity, **B** PROMIS pain intensity, **C** BPI pain interference, **D** PROMIS pain interference, **E** Urinary score of USSQ (USSQ-U), **F** LURN SI-10 urinary symptoms

Daily assessments of pain intensity, pain interference, and urinary symptoms are displayed in Fig. 1. The percentage of completed questionnaires was very high, ranging from 84.6% to 98.8% completion on any given day. Similar patterns in daily pain severity were seen using both BPI and PROMIS. Pain intensity increased from baseline to POD 1, remained elevated over the duration of the stent, and varied widely among individuals. Median pain intensity scores were highest during the first 2 days after URS, with variable changes thereafter, but remained persistently elevated compared to baseline.

Pain interference was noted to have a similar pattern as pain intensity early in the postoperative course. Pain interference scores increased versus baseline on POD 1, with diminution over time, approaching baseline after POD 5. As seen with pain intensity, there was also wide inter-participant variation in reported interference due to pain.

Urinary symptoms increased from baseline and appeared to peak the day after surgery as measured by both instruments. Urinary symptoms remained persistently elevated versus baseline, without substantial decrease until after stent removal. Finally, bother due to urinary symptoms increased and was most pronounced immediately after URS. While median bother score mirrored the baseline value by POD 3, there was not definitive resolution until after stent removal. Wide variation among individuals was again noted.

## Discussion

This prospective cohort study was the first to measure the daily lived experiences of patients after URS for stone disease. We found that there was wide variation of self-reported pain and urinary symptoms daily, stent symptoms peaked within the first 2 days after surgery but remained elevated throughout stent dwell time, and interference with daily activities secondary to pain persisted longer compared to bother due to urinary symptoms. We also found consistency among various instruments that assessed similar concepts. These results inform the selection of times at which stent symptoms should be measured after URS and, given the variation in symptoms among participants, reveal the importance of identifying characteristics that may help understand which patients are at greatest risk of severe stent symptoms.

Most importantly, this study identified days of the most intense SAS following URS. This information is important in determining the days at which SAS should be measured in research studies and also for counseling patients about the expected experience after URS. We found that the magnitude of adverse symptoms occurred within the first 2 days, a finding consistent among various instruments we used. This is in accord with a previous report by Lingeman and colleagues, who suggested that symptoms peaked on the first day based on pain medication use alone, although stent symptoms were not formally measured until POD 4 [12]. Our findings from daily assessment of SAS informed decisions for the main STENTS cohort. We chose POD 1, 3, and 5 (in addition to day of stent removal and 30 days after removal) in order to capture the peak symptoms and further analyze the trajectory of symptoms after URS. Our results indicate that there is no definitive time point in which symptom assessment is not relevant to measure, since the symptoms persist until stent removal.

The substantial variation in the reported pain intensity, pain interference, urinary symptoms, and urinary bother among participants across our study period highlights the heterogeneity in the experience of individuals undergoing URS for stones. To date, little is known about risk factors for severe SAS, and further investigation is needed. Younger age has been reported to be associated with more severe pain and unplanned hospital visits [13–15]. Krambeck and colleagues, in a subset analysis, reported decreased analgesic use in young males who had a ketorolac-loaded stent, suggesting potential gender differences in stent tolerance [16]. In our study, while bother due to urinary symptoms was most pronounced on POD 1 and 2, median bother was similar to baseline on subsequent days. In a recent study comparing stent designs, Wiseman and colleagues postulated that the effect of surgery on urinary symptoms may improve sooner than pain [17]. A detailed investigation of the effects of patient characteristics, stone factors, operative instrumentation, and medication use on SAS is the aim of the full STENTS study, which will elucidate the potential causes of SAS heterogeneity, identify patients at highest risk for severe SAS, and may allow for the creation of a prediction model [7], all of which would be useful in counseling patients in preemptive therapeutic decision-making and identifying populations to study in future trials.

An important finding of this study is that there was consistency observed among instruments that assessed similar domains of the patient experience. We intentionally chose to perform a comprehensive assessment of SAS using various instruments with conceptual overlap. For example, pain intensity and pain interference were measured using the BPI and PROMIS. Pain interference refers to pain that limits the patient’s ability to engage in daily activities, such as social-, work-, or school-related functions. BPI has been used in pain-related studies widely, contains a complete body map, and measures both the intensity of pain as well as the interference of pain in one’s life. This is the first study, to our knowledge, to use BPI following URS and stent placement. Pain interference was seen to approach baseline after several days as measured by the BPI. The potential significance of this finding is unclear, but there could be some element of adjustment to SAS despite continuing to have pain, as measured by pain severity. Pain intensity and interference were also measured with individual PROMIS instruments, allowing for comparison with population norms. Recently, PROMIS measures have been used to characterize stone patients in various settings [18–20]. In our study, we noted consistency among instruments in ascertaining peak symptoms, the evolution of daily symptoms, and the heterogeneity of the lived experiences following URS and stent placement.

Our study is the first to determine the daily pattern of pain intensity, pain interference, urinary symptoms, and bother after URS with stent placement for the entire postoperative period; however, the following limitations indicate opportunities to build on these findings. The large interindividual variation in symptoms coupled with a relatively small sample size precluded making multiple comparisons of the different domains of pain intensity, pain interference, urinary symptoms, and bother. These comparisons will be addressed in the main STENTS cohort. Additionally, participants were recruited from four clinical centers, and treatment of SAS post-URS differed among urologists. Lastly, we did not consider clustering by surgeon or institution.

## Conclusion

In this first study measuring the daily lived experiences of patients after URS for stone disease, daily experiences of pain intensity, pain interference, urinary symptoms, and bother were highly variable but seemed to peak in the first 2 days and remained elevated while the stent was in place. These findings may inform more counseling of patients, shared decision making, and serve as groundwork to facilitate additional research for evaluating therapies and understanding mechanisms and risk factors for adverse experiences after ureteroscopy.

## Supplementary Information

**Additional file 1**. USDRN members and affiliations.

## Data Availability

Request for data and materials will be reviewed and addressed according to the network’s policy for ancillary studies. Requests can be e-mailed to USDRN@dm.duke.edu.
