# Supplementary material for: Quality of life impact and recovery after ureteroscopy and stent insertion: insights from daily surveys in STENTS
Source: BMC Urol. 2022 Apr 6;22:53. doi: 10.1186/s12894-022-01004-9 (PMC8988384; doi:10.1186/s12894-022-01004-9)
Supplement: Supplementary file 1 — Additional file 1. USDRN members and affiliations. [file 12894_2022_1004_MOESM1_ESM.docx]

**NIDDK Urinary Stone Disease Research Network Contributors**

**Urinary Stone Disease Research Network**: The following individuals were instrumental in the planning and conduct of the STENTS study at each of the participating institutions:

*Clinical Centers*

University of Pennsylvania/Children’s Hospital of Pennsylvania, Philadelphia, PA: PI: Peter P Reese, MD, MSCE, Gregory E. Tasian, MD MSCE, CoIs: Justin Ziemba, MD MSEd. Study Coordinators: Emily Funsten, Adam Mussell, Rebecca McCune, Salima Shah, Arushi Jain, Antoine Selman-Fermin.

University of Texas Southwestern Medical Center, Dallas, TX: PI: Naim M. Maalouf MD, Jodi A. Antonelli MD. Co-I: Brett A. Johnson MD, Margaret S. Pearle, MD PhD, Linda A. Baker MD. Study Coordinators: Brooke Piskator, Joyce Obiaro, Cynthia Rangel, Martinez Hill.

University of Washington, Seattle, WA: PI: Jonathan D. Harper, MD, Hunter Wessells, MD; Co-I: Michele Curatolo MD, Todd Edwards PhD, Mathew Sorensen MD, Collaborator: Robert Sweet, MD. Study Coordinators: Tristan Baxter, Holly Covert, Elsa Ayala, Lisa Flint.

Washington University in St. Louis, St. Louis, MO: PI: Alana C. Desai, MD, H. Henry Lai, MD; Co-Is: Kefu Du, MD; Study Coordinators: Susan Mueller RN, Linda Black RN, Aleksandra Klim RN.

*Scientific Data Research Center*

Duke Clinical Research Institute, Duke University, Durham, NC: PI: Charles D. Scales, Jr MD MSHS, Hussein R. Al-Khalidi PhD. Co-I: Amy Corneli, PhD, Bryce Reeve, PhD, Kevin Weinfurt PhD. Statistician: Honqiu Yang, PhD.  STENTS Project Lead: Davy Andersen. USDRN Project Lead: Laura Johnson. Lead CRA: Andrew Dodd. Data Manager: Omar Thompson. Qualitative Research Core: Carrie Dombeck, Kevin McKenna, and Teri Swezey.

*National Institute of Diabetes and Digestive and Kidney Diseases (NIDDK)*

Project Scientist: Ziya Kirkali, MD; Program Official: Christopher Mullins, PhD.

Funding:

DK110986 (WUSTL)

DK110961 (CHOP/UPENN)

DK110954 (UW)

DK110995 (UTSW)

DK110988 (Duke)

This is a cooperative agreement; that means there is substantial federal scientific or programmatic involvement in the research activities. The NIDDK Project Scientist (ZK) is involved in the design and development of the clinical protocol, preparation of questionnaires and other data recording forms, coordination of research, statistical evaluations and analyses of data, and the publication of results. The program is overseen by an independent NIDDK Program Official.
